# The Epidemiology and the Treatment of Fractures Due to Electric Scooters: A Comparison of Pediatric and Adult Age Groups

**DOI:** 10.7759/cureus.37807

**Published:** 2023-04-18

**Authors:** İsmail Büyükceran, Ahmet Ersoy, Cahit Şemsi Şay, Hüseyin Sina Coşkun, Yılmaz Tomak

**Affiliations:** 1 Department of Orthopedics and Traumatology, Ondokuz Mayıs University, Samsun, TUR

**Keywords:** trauma pediatric, extremity fracture, electric scooter accidents, orthopedic trauma, injury

## Abstract

Aim: Electric scooters (e-scooters) are widely used as alternative vehicles worldwide. These light vehicles do not need a license to drive, and it is also popular among vehicles under the age of 18 among users in Turkey. This is a new term in the literature since there is an increase in accidents resulting from this overuse. This study aims to reveal the patterns and severity of orthopedic injuries resulting from using an e-scooter, especially in the pediatric population.

Patients and methods: Patients who were admitted to the university hospital emergency department due to using an e-scooter and had fractures were retrospectively analyzed. Demographic data, admission times, injury mechanisms, and fracture patterns of the patients were documented.

Results: Forty-nine (49.4%) of 99 patients were under 18, and 50 (50.6%) were over 18. It is seen that 58.5% (58 patients) had an accident by falling spontaneously, 37.3% (37 patients) collided with vehicles in traffic, and 4.2% had an accident by colliding with a standing object. While 59.5% of the upper extremity fractures are seen, 27.2% are lower extremity fractures. Multiple fractures were observed in 13.3%.

Conclusion: Pediatric population frequently uses these alternative means of transport. The pediatric group usually had upper extremity injuries, while adults mostly had lower extremity injuries. Care must be taken when children are drivers of e-scooters.

## Introduction

Technological advances are rapid in transportation, as in every field. With the impact of technological developments on society, alternative transportation models have begun to emerge [1,2]. There has been an increase in electric scooters (e-scooters) as easy transportation devices worldwide. First popularized in 2001 by Segway, personal transporters were initially expensive, restricted to a few professions, and associated with specific injuries [3-5]. Since its launch in 2017, e-scooters have started to be used in major cities as they offer solutions to a wide range of transportation policy objectives and are easy to reach [6,7].

The market for affordable, personal stand-up e-scooters in busy cities has completely changed the demographics of accident-related injuries [8,9]. In addition to Turkey, there has been an increase in the use of e-scooters as easy transportation devices worldwide [7]. With the increase in the use of e-scooters, accidents and injuries have increased in direct proportion. The most frequent injuries included fractures, head injuries, isolated contusions, sprains, and lacerations [10]. Although the data on the increase in musculoskeletal injuries due to the short launch time of e-scooters are scarce, publications from the USA and Germany prove this increase [11].

Although the data on the increase in musculoskeletal injuries due to the short launch time of e-scooters are scarce, existing literature indicates that e-scooters can cause severe musculoskeletal injury and are typically associated with high-energy trauma [11,12].

This study aims to demonstrate a new source of pediatric trauma and lead attention to using these light vehicles. Also, this study intended to determine if there is a difference between the adults and the pediatric population regarding orthopedic injuries while using these vehicles.

## Materials and methods

This study retrospectively evaluated all injuries admitted to the emergency department between 01.01.2019 and 30.10.2022 and used an e-scooter as a driver. Injuries caused by vehicles such as bicycles and light motorcycles are excluded. Age and gender distribution, type and mechanism of injury, time of admission, type of injury, and trauma score (ISS) were collected. The patients are divided into two groups: Group 1 (pediatric group) was formed by patients under 18, while Group 2 (adult group) was formed by patients above 18.

By examining the age and gender distribution, it was predicted that the excess of e-scooter use under the age of 18 and the incidence of fractures due to this increase. The admission time to the hospital was divided into 6-hour periods and examined as 24.00-06.00, 06.00-12.00, 12.00-18.00, and 18.00-24.00.

Statistical analysis

Data obtained in the study were analyzed statistically using SPSS for Windows 21.0 software (SPSS Inc, Chicago, IL, USA). Descriptive statistics were presented as mean ± standard deviation, median (minimum-maximum) values, frequency (n), and percentage (%). Descriptive analyzes were performed. A chi-square test was performed for damage zones according to accident mechanisms. P value <0.05 has been considered significant. The chi-square test was used when comparing fracture patterns according to age distributions. Ethical committee approval was obtained from the institutional board.

## Results

The study consists of 99 patients. It is seen that 49 (49.4%) of these patients were under the age of 18, and 50 (50.6%) were over the age of 18. There were 49 male and 50 female patients. A total of 58.5% (n=58) had an accident by falling spontaneously, 37.3% (n=37) collided with vehicles in traffic, and 4.2% (n=4) had an accident by colliding with a standing object. A total of 31.3% of the patients (31 patients) had an accident between 00.00 and 06.00 and were admitted to the emergency room, while 14.1% were 12.00-18.00 and 54.6% were between 18.00 and 24.00. Table 1 details all demographic data, accident mechanisms, and accident occurrence times.

**Table 1:** Demographic data of 99 patients who had an accident with an e-scooter *p value <0.05 is significant ISS: Injury Severity Score

		Group 1 (Pediatric Group)	Group 2 (Adult Group)	P value (<0.05)*
AGE		49 (4-17)	50 (18-80)	
SEX	Male	29	20	
	Female	31	19	
ACCIDENT MECHANISM	Spontaneous fall	32	26	0.08
	Striking a moving object	17	20	
	Hitting a standing object	0	4	
TIME OF ACCIDENT	00.00-06.00	13	18	0.57
	06.00-12.00	0	0	
	12.00-18.00	7	7	
	18.00-24.00	29	25	
ISS SCORE	Over 15	11	10	0.76
	Under 15	38	40	
Total		49 (100%)	50 (100%)	

Age and accident hours were compared between the groups, and no significant difference was found (p=0.057). There was no significant difference between the groups regarding accident mechanism, time of the accident, and ISS scores.

Injury localizations and mechanisms of injuries are summarized in Table 2. As seen in Table 2, when the injured areas were compared according to accident mechanisms, patients who fell spontaneously had mostly upper extremity fractures, while patients who had an accident with vehicles in traffic and hit a stationary object had more fractures in the lower extremities, and it was found statistically significant (p=0.001).

**Table 2:** Distribution of injury zone according to accident mechanism

Mechanism of Injury		Injury Localization			
		Upper Limb	Lower Limb	Both	P value*
	Spontaneous fall	48	10	0	0.001
	Striking a moving object	9	15	13	
	Hitting a standing object	2	2	0	
Total		59	27	13	

When the fracture patterns of patients younger than 18 and older are compared, more clavicle and humerus fractures are seen in patients younger than 18. In comparison, more radial and tibia fractures are seen in patients over 18 years of age (p=0.00) (Table 3).

**Table 3:** Classification of fracture patterns under 18 years old and above

Fracture Pattern	Age<18 years	Age>18 years	P value
Clavicula	16	4	
Humerus	7	2	
Elbow	4	0	
Radius	7	18	
Femur	2	5	
Tibia	7	13	
Hand	1	7	
Multiple sites	5	1	
Total	49	50	0000

## Discussion

E-scooters have gained popularity in recent years due to their easy accessibility, their fast and economic advantages, and also allow social distancing. The number of users is increasing day by day. With the increase in the number of users, accidents and health expenditures are also among the possible consequences [13].

Our study shows that most people using e-scooters are under 18. As a result of injuries, patients under 18 experience disruption in their education and difficulty meeting their daily needs. Bloom et al., in their study, only 3% of people using e-scooters in Los Angeles reported using helmets and protective equipment [14].

Paudel et al. emphasize that the center of gravity changes forward and upwards due to the driver standing, sudden deceleration with the e-scooter, or impact [15]. They report that upper extremity injuries are seen more frequently due to the center of gravity change. Our study found it statistically significant that upper extremity fractures due to the falling mechanism were more common.

**Figure 1:**
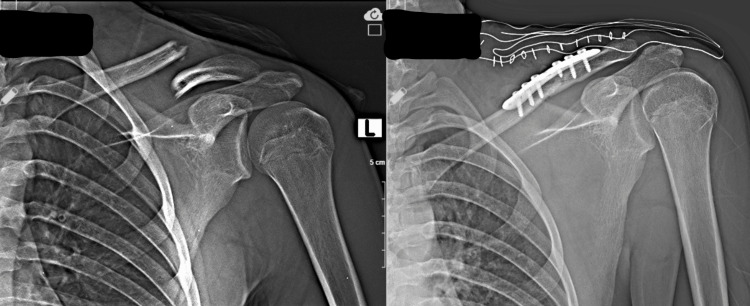
Fifteen-year-old male falls from an e-scooter. The plain radiograph on the left shows a displaced clavicle shaft fracture. Treatment has been made as open reduction and internal fixation. The post-operative radiograph is given on the right.

The treatment modalities vary according to the fracture pattern and the mechanism of occurrence. Only two patients underwent open reduction internal fixation in clavicle fractures due to skin irritation (Figure 1).

**Figure 2:**
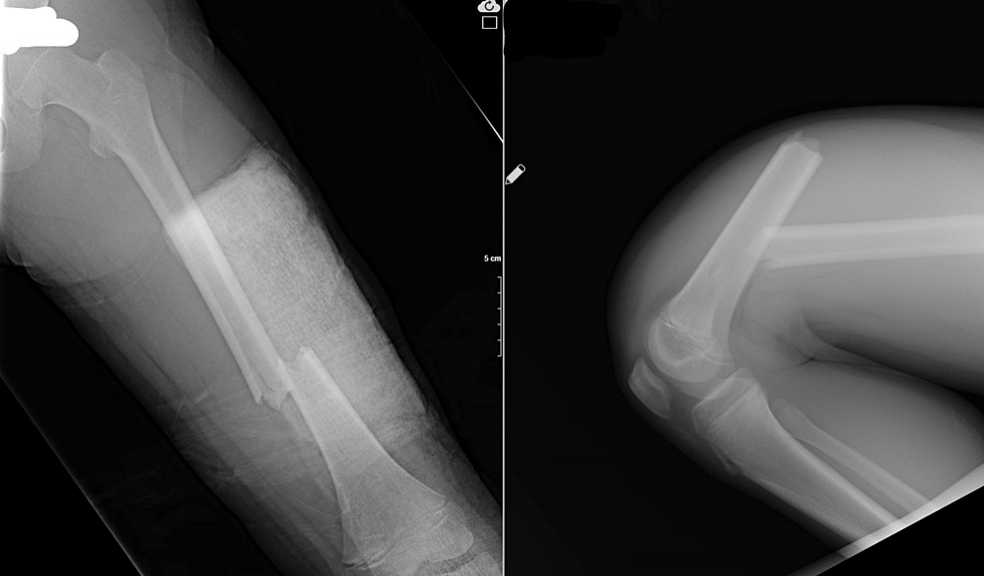
Fifteen-year-old male displaced left femur shaft fracture

**Figure 3:**
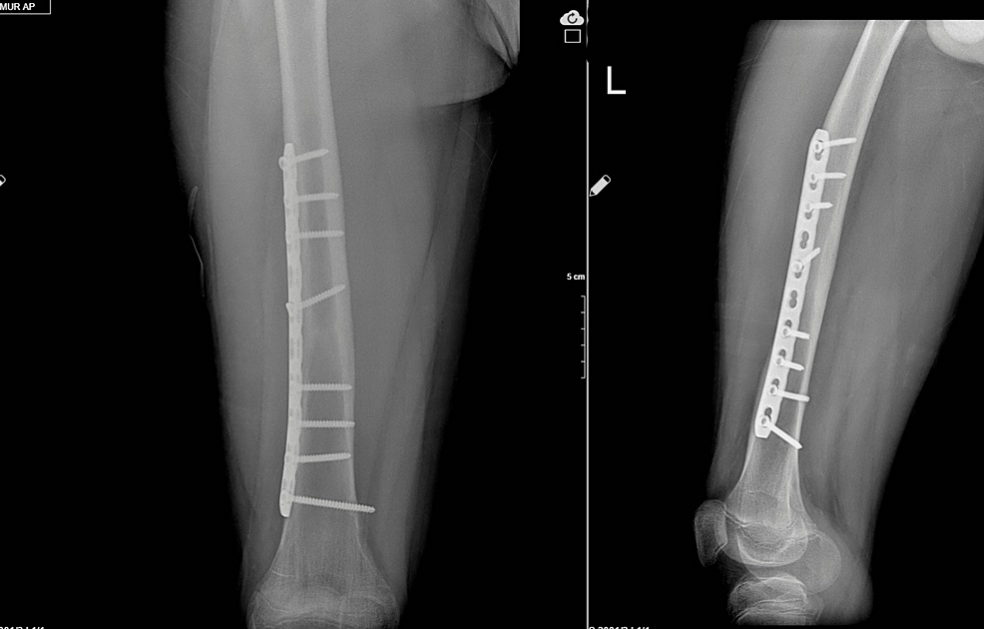
Treated with plate-screw osteosynthesis. Six months follow-up radiographs show the union of the fracture site.

All other clavicle fractures have been followed conservatively. The fractures of nine patients with humeral fractures were non-displaced fractures suitable for conservative follow-up, so they were followed up conservatively. All four patients with elbow fractures were under 18 and operated on because of displaced fractures involving the joint, and no complications were observed during their follow-up. Lower extremity fractures were usually treated operatively, and early range of motion was encouraged (Figure 2 and Figure 3).

Although six patients had fractures in multiple regions, seven of the 33 lower extremity fractures were open. Appropriate antibiotic therapy was administered after the temporary fixation of open fractures with an external fixator. After excluding the risk of infection, their definitive surgical treatment was made, and no complications were observed in the follow-up. Usually, open fractures had a high tendency to produce complications. We believe these complications are avoided because of the early administration of antibiotherapy and adequate debridement.

The financial burden is a big concern in this new orthopedic trauma requiring surgical intervention. A current study in the literature stated that e-scooter-related injuries in the employed population had caused a substantial economic impact, with more than 1,000,000€ the indirect cost of our hospital during the reviewed period [16]. However, we can not state a financial issue in this article. This is a subject of further study.

The study has several limitations. First, this study was conducted with a limited number of patients, as it was retrospectively designed and conducted in a single center. Therefore, it covers a small part of the e-scooter-related orthopedic injury model. Secondly, long-term follow-ups of patients are needed to understand better the burden that e-scooter has created for the economic and health systems. Thirdly, no comparison could be made in our study because the groups were heterogeneous regarding wearing helmets or protective equipment. It cannot be said in this context that wearing protective equipment reduces injuries. Furthermore, the last limitation, patients who were admitted to the emergency department due to an injury while using an e-scooter and who had fractures, were included. Patients with minor injuries from the e-scooter are not included in the study.

## Conclusions

Increasing the use of e-scooter as an alternative vehicle may cause orthopedic problems. These types of injuries affect the pediatric population and adults. The pediatric population is usually affected in the upper extremity, while adults suffer from lower extremity fractures. Public education and social awareness can prevent these injuries and workforce loss.
